# Site-selective growth of surface-anchored metal-organic frameworks on self-assembled monolayer patterns prepared by AFM nanografting

**DOI:** 10.3762/bjnano.4.71

**Published:** 2013-10-11

**Authors:** Tatjana Ladnorg, Alexander Welle, Stefan Heißler, Christof Wöll, Hartmut Gliemann

**Affiliations:** 1 Institute of Functional Interfaces (IFG), Karlsruhe Institute of Technology (KIT), Hermann-von-Helmholtz-Platz 1, 76344 Eggenstein-Leopoldshafen, Germany; 2 Institute for Biological Interfaces 1 (IBG1), Karlsruhe Institute of Technology (KIT), Hermann-von-Helmholtz-Platz 1, 76344 Eggenstein-Leopoldshafen, Germany

**Keywords:** atomic force microscopy (AFM), metal-organic frameworks, nanografting, nanoshaving, SURMOF

## Abstract

Surface anchored metal-organic frameworks, SURMOFs, are highly porous materials, which can be grown on modified substrates as highly oriented, crystalline coatings by a quasi-epitaxial layer-by-layer method (liquid-phase epitaxy, or LPE). The chemical termination of the supporting substrate is crucial, because the most convenient method for substrate modification is the formation of a suitable self-assembled monolayer. The choice of a particular SAM also allows for control over the orientation of the SURMOF. Here, we demonstrate for the first time the site-selective growth of the SURMOF HKUST-1 on thiol-based self-assembled monolayers patterned by the nanografting technique, with an atomic force microscope as a structuring tool. Two different approaches were applied: The first one is based on 3-mercaptopropionic acid molecules which are grafted in a 1-decanethiolate SAM, which serves as a matrix for this nanolithography. The second approach uses 16-mercaptohexadecanoic acid, which is grafted in a matrix of an 1-octadecanethiolate SAM. In both cases a site-selective growth of the SURMOF is observed. In the latter case the roughness of the HKUST-1 is found to be significantly higher than for the 1-mercaptopropionic acid. The successful grafting process was verified by time-of-flight secondary ion mass spectrometry and atomic force microscopy. The SURMOF structures grown via LPE were investigated and characterized by atomic force microscopy and Fourier-transform infrared microscopy.

## Introduction

**Figure 1:**
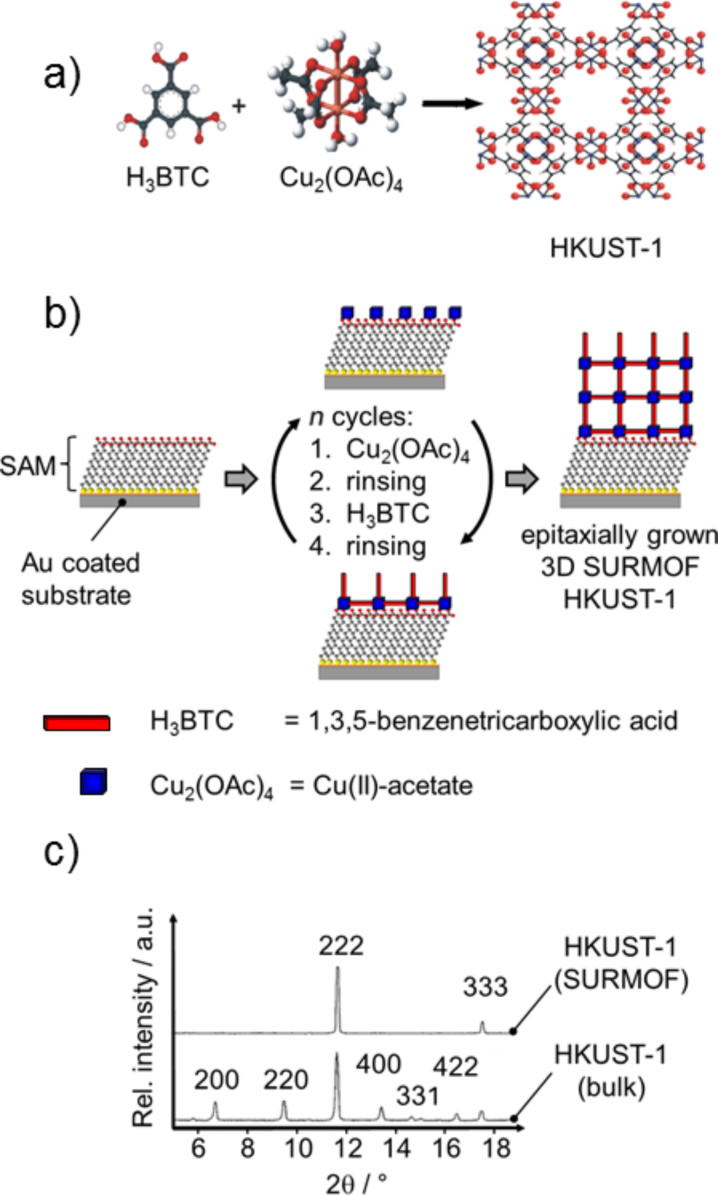
(a) Building units for the growth of MOF HKUST-1 and the unit cell of HKUST-1. (b) The principle of the layer-by-layer growth of a surface anchored MOF. (c) XRD results for a polycrystalline particulate HKUST-1 sample and the corresponding SURMOF. The oriented growth of the SURMOF material is clearly proven by the two reflexes of the SURMOF in the [111] direction [5].

Metal organic frameworks (MOFs) are highly crystalline three-dimensional micro- and mesoporous materials that consist of metal ions or metal-oxo units (serving as nodes) interconnected by organic linkers. In conventional synthesis the MOFs are formed in a solvothermal process, and the reaction products precipitate in the form of crystalline powders [1–2]. One of the best-known MOFs is HKUST-1, first introduced by Chui et al. [3]. This MOF consists of “paddle wheels” formed by attaching 1,3,5-benzenetricarboxylate linkers to a Cu^2+^-dimer (see Figure 1a). Meanwhile several thousands of different MOF structures are documented in the literature [4]. The high variety of the nodes and linkers as well as the huge number of possible combinations allows, in principle, the preparation of an almost infinite number of different MOF structures with different chemical and/or physical and geometrical properties (e.g., pore size and pore structure, etc.), which can be tailored for the corresponding application.

**Figure 2:**
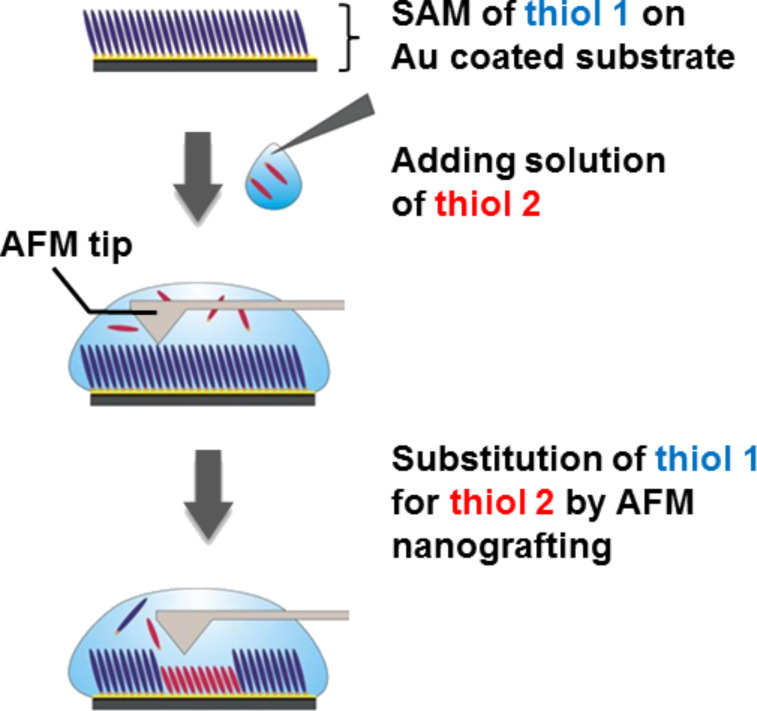
Scheme of the nanografting process.

As a particulate system this class of material is already applied in the field of nanotechnology, e.g., for gas storage and gas separation [6–8], catalysis [9], delivery of therapeutic agents [10–12] and sensor devices [13]. Presently, more advanced applications are discussed, in particular in medicine and technology. For many of the more advanced applications the MOF materials need to be deposited on a solid substrate. Several methods have been introduced to create MOF-based coatings (for a recent review see [14]). One possible procedure is the so called in-situ synthesis, which was introduced by Bein et al. [15]. In this synthesis the MOF crystals are grown by dipping a gold coated substrate, which is terminated with a thiol-based self-assembled monolayer (SAM), into a solution containing a mixture of the metal nodes and the organic linkers. As a result, the MOF crystal growth is started by a substrate-induced nucleation process. Many of the methods developed to deposit MOFs on solid substrates suffer from the fact that the resulting MOF layer has a polycrystalline character due to the random orientation of the crystals on the substrate. In the field of catalysis, for example, this polycrystalline character might hinder or even suppress the free diffusion of molecules or reaction products into or out of the MOF layer. To overcome these limitations, which result from the highly polycrystalline nature of the MOF-coatings, a novel method was recently developed that produces very smooth, homogeneous MOF-coatings. These surface anchored metal–organic frameworks (SURMOFs) exhibit a uniform layer thickness and are fabricated using a novel layer-by-layer (LBL) method [16–20]. This procedure is schematically shown in Figure 1b. First, a gold coated substrate is modified by the deposition of a thiol-based SAM, that carries either an –OH, a –COOH or a pyridine unit. The SAM, and in particular its surface termination, plays a crucial role in this context and also determines the growth direction of the SURMOF [14–15]. On a CH_3_-terminated SAM no deposition takes place [16]. Subsequent to SAM deposition the substrate is immersed into a solution of the metal node, rinsed, dipped in the linker solution and rinsed again. After this procedure the first layer of the SURMOF is deposited and the SURMOF thickness can be precisely and reproducibly controlled by the number of cycles [21]. The size of the pores within this crystalline periodic material can be controlled by the length and the size of the organic ligands [22–24]. As SURMOFs grow in an oriented way and the layers are oriented parallel to the substrate molecules can diffuse from the surface of the SURMOF to the supporting substrate without hindrance. Therefore, SURMOFs can be used as model systems to gain a detailed understanding of, e.g., the kinetics of sorption/desorption processes or as active sites for sensor systems [16]. The high degree of orientation and the high structural quality of a SURMOF are evidenced by the XRD data. Figure 1c shows a comparison of recorded data of a polycrystalline powder MOF and of the corresponding SURMOF, both for the case of HKUST-1. While the powder MOF shows all reflexes of different orientations of the crystal, in the case of the SURMOF produced by the LBL method, in the out-of-plane XRD data only the [111] direction is detected. This is a direct proof that the LBL preparation results in a highly oriented SURMOF with (111)-layers grown parallel to the surface. A particular advantage of using thiol-based SAMs as a templating surface for the deposition of SURMOF structures on solid substrates is the availability of a large number of processes for the lateral structuring of SAMs, e.g., micro contact printing (µCP) [25]. With the application of these methods the selective deposition of SURMOFs is straightforward, as was shown recently for the case of HKUST-1 grown on a 16-mercaptohexadecanoic acid- (MHDA-) based SAM pattern created by µCP [18,21]. The feature sizes that can be achieved with this method, however, are limited and the preparation of sub-micrometer sized patterns is challenging or even impossible for µCP. The fabrication of structures within SAMs [26] of higher resolution can be obtained by nanoshaving and nanografting [27] or other methods based on scanning probe microscopy techniques, e.g., atomic force microscopy (AFM) [28–29]. Both lithography methods allow for lateral structuring with resolutions down to several nanometers. They are based on the cleavage of the bond between the thiolate species and the Au-substrate by an AFM tip. Depending on the applied force this cleavage can be carried out without scratching the substrate. Another advantage of the nanoshaving technique is the possibility to perform it in liquid or in air, depending on the system which should be modified. Nanoshaving in air or in a clean solvent always results in a removal of defined molecules [30] while nanografting [27] is usually performed in an organothiol containing solution, enabling the site-selective substitution of the removed SAM molecules by other SAM molecules which are present in the solution (Figure 2). The use of nanografting and nanoshaving for the fabrication of nanometer-sized structures, which consist of MOF material will be of interest for many applications. An area of potential interest are biological studies. It is known that cell adhesion depends strongly on both the chemical functionalization of the surface as well as on the distribution of the chemical functionalities [31–32].

In this work we demonstrate the successful growth of micrometer sized HKUST-1 structures on patterns inside thiolate-based SAMs by using the AFM as nanografting tool. ToF-SIMS analyses were carried out to demonstrate the successful SAM patterning by nanografting, while FT-IR microscopy and AFM were used to verify the SURMOF growth on the patterned substrate.

## Results and Discussion

The in-situ nanografting process includes two processes: (1) shaving of the original SAM made from the first thiol followed by (2) the refill of the shaved areas with the second thiol, which occurs instantaneously during the shaving as the shaving is carried out in a solution of the second thiol. Before doing the actual grafting, the parameters for the nanografting process (vertical force applied by the AFM tip to the substrate, scan speed, etc.) were optimized.

### HKUST-1 SURMOF on MPA nanografted structures

**Figure 3:**
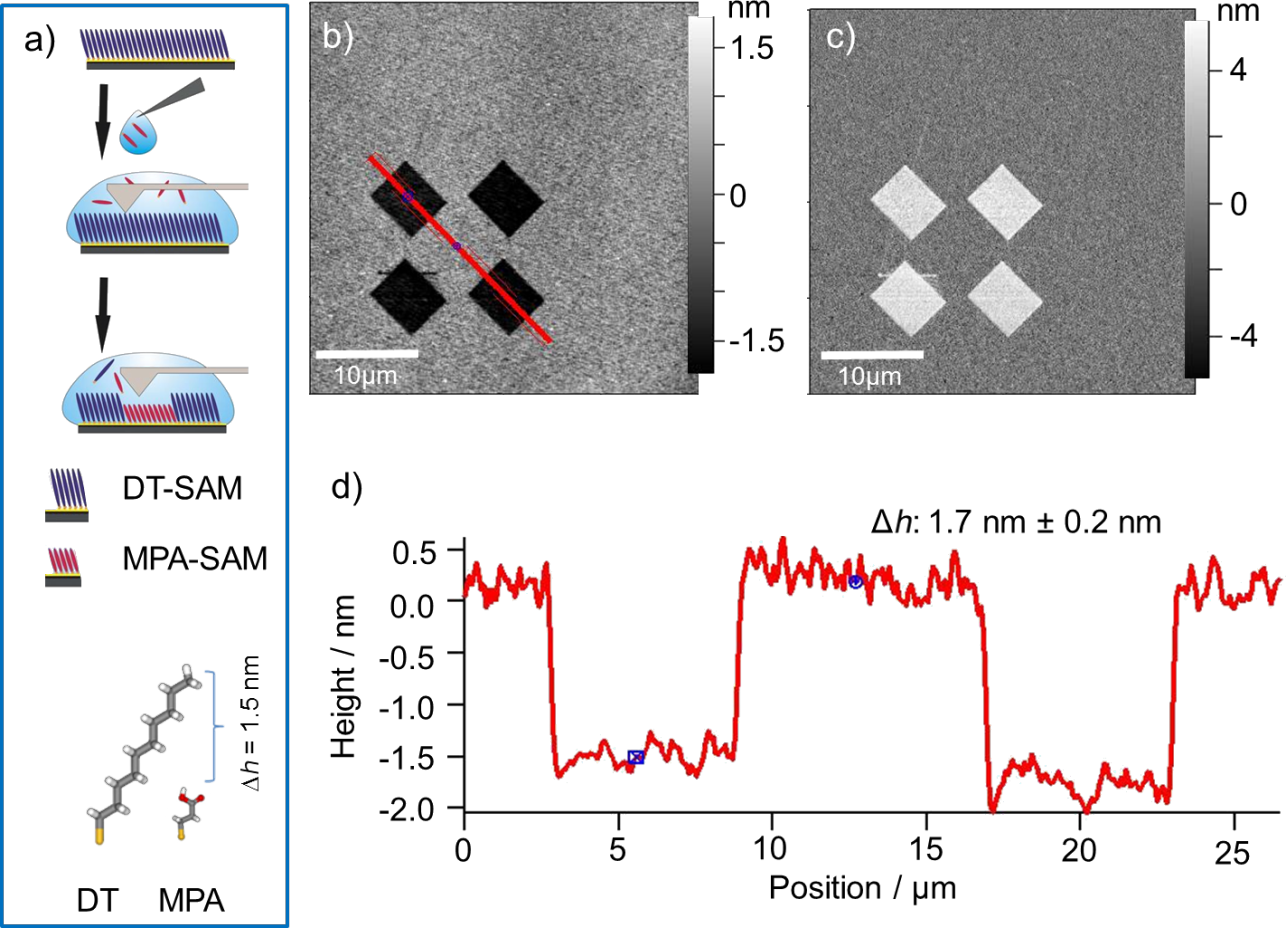
(a) Scheme of the grafting experiment and used SAM molecules (without H-atoms). AFM-topography (b) and -phase (c) image of MPA structures grafted in a DT SAM matrix. (d) Cross section along the red line in (b).

To obtain a patterned deposition of HKUST-1, nanografting was first carried out within a SAM matrix made of 1-decanethiol (DT). A pattern was created by shaving with the AFM tip and the removed 1-decanethiolates were substituted by 3-mercaptopropionic acid (MPA) molecules present in the supernatant ethanol (Figure 3a). Figure 3b and Figure 3c show the AFM-topography and AFM-phase images, respectively, immediately after the areas of four MPA rectangles were grafted into the DT matrix SAM with the AFM tip. The height difference between the DT SAM and the grafted MPA coated features was determined from the cross section in Figure 3d along the red line in Figure 3b. The measured height difference of 1.7 ± 0.2 nm is in good agreement with the theoretical length difference between the two thiols (DT and MPA) of 1.5 nm. In addition, the phase contrast data shown in Figure 3c reveal the presence of two different materials inside and around the grafted areas. These findings directly demonstrate the success of the grafting experiment.

**Figure 4:**
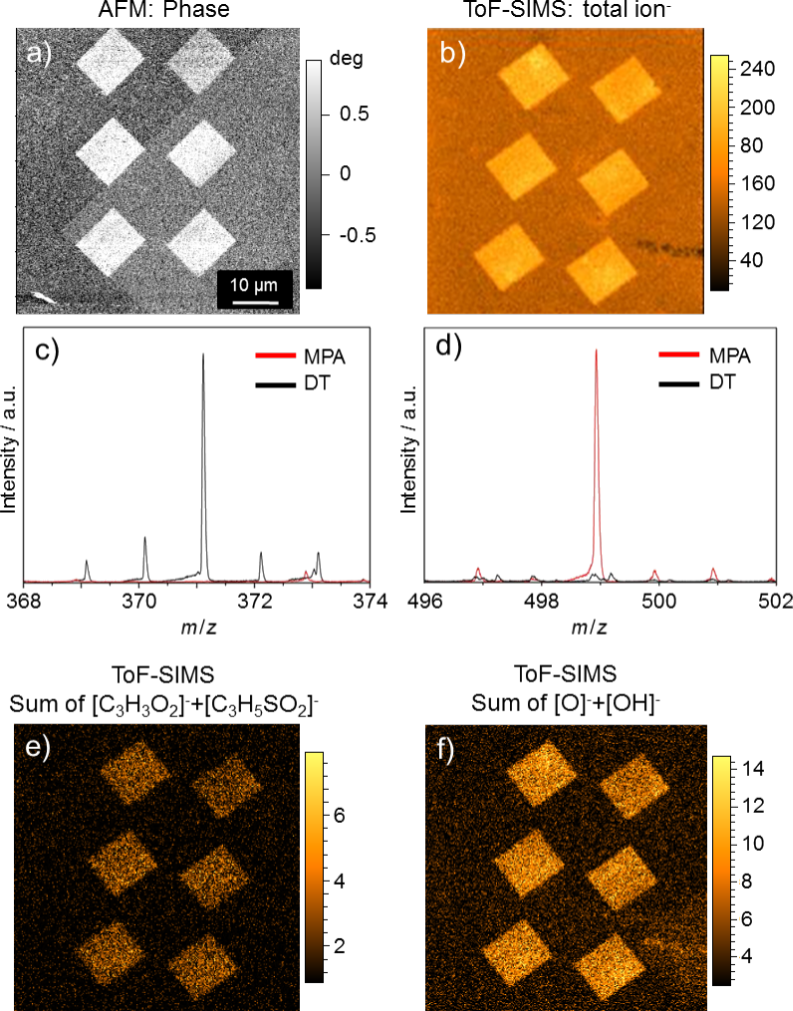
(a) AFM phase contrast image of six rectangles consisting of MPA grafted into a DT matrix SAM. (b) Negative polarity total secondary ion mapping. (c) High mass resolution SIMS data for DT (black line) and MPA (red line), the peak at *m*/*z* 371 in (c) is attributed to [AuSC_10_H_22_]^−^, the Au cluster ion of DT, (d) as (c) the peak at *m*/*z* 499 is assigned to [Au_2_SC_3_H_5_O_2_]^−^, the Au cluster ion of MPA. Other characteristic peaks are found at *m*/*z* 105, [C_3_H_5_SO_2_]^−^, and *m*/*z* 301, [AuSC_3_H_4_O_2_]^−^. (e) Local distribution of MPA fragments [C_3_H_3_O_2_]^−^ and [C_3_H_5_SO_2_]^−^, (f) local distribution of [O]^−^ and [OH]^−^.

In addition to the AFM investigations, which provided information about topography and material contrast of the grafted sample, a chemical characterization was carried out by time-of-flight secondary ion mass spectrometry (ToF-SIMS). For that purpose a sample with grafted rectangular structures of 10 µm × 12 µm was prepared. The corresponding AFM-phase image is shown in Figure 4a. According to the results in Figure 3c, the grafted rectangular MPA coated structures show a significantly different phase shift compared to the 1-decanethiolate matrix SAM. The result of the negative polarity total secondary ion mapping is shown in Figure 4b. High mass resolution spectra, obtained with very short Bi^+^ primary ion pulses (1 ns), “high current bunched mode”, allowed for the detection of several very characteristic cluster ion peaks. Most importantly, the corresponding Au cluster ion of DT [AuSC_10_H_22_]^−^ at *m*/*z* 371 (Figure 4c) and the Au cluster ion of MPA [Au_2_SC_3_H_5_O_2_]^−^ at *m*/*z* 499 (Figure 4d) were detected as characteristic peaks. While this spectrometry mode allows for an unambiguous chemical assignment, the lateral resolution of the analysis is limited due to a primary ion beam spot diameter of approx. 5 µm. To obtain higher lateral resolutions, imaging was performed with a non-bunched primary ion pulse without chromatic aberration, thus providing nominal mass resolution. This analysis showed the expected pattern of MPA fragments, e.g., [C_3_H_3_O_2_]^−^ and [C_3_H_5_SO_2_]^−^ (Figure 4e). As strong peaks of fragmented ions provide the highest contrast, the sum of O^−^ and OH^−^ secondary ions is shown in Figure 4f which was produced by imaging 256 × 256 pixels in a 65 µm × 65 µm field of view.

**Figure 5:**
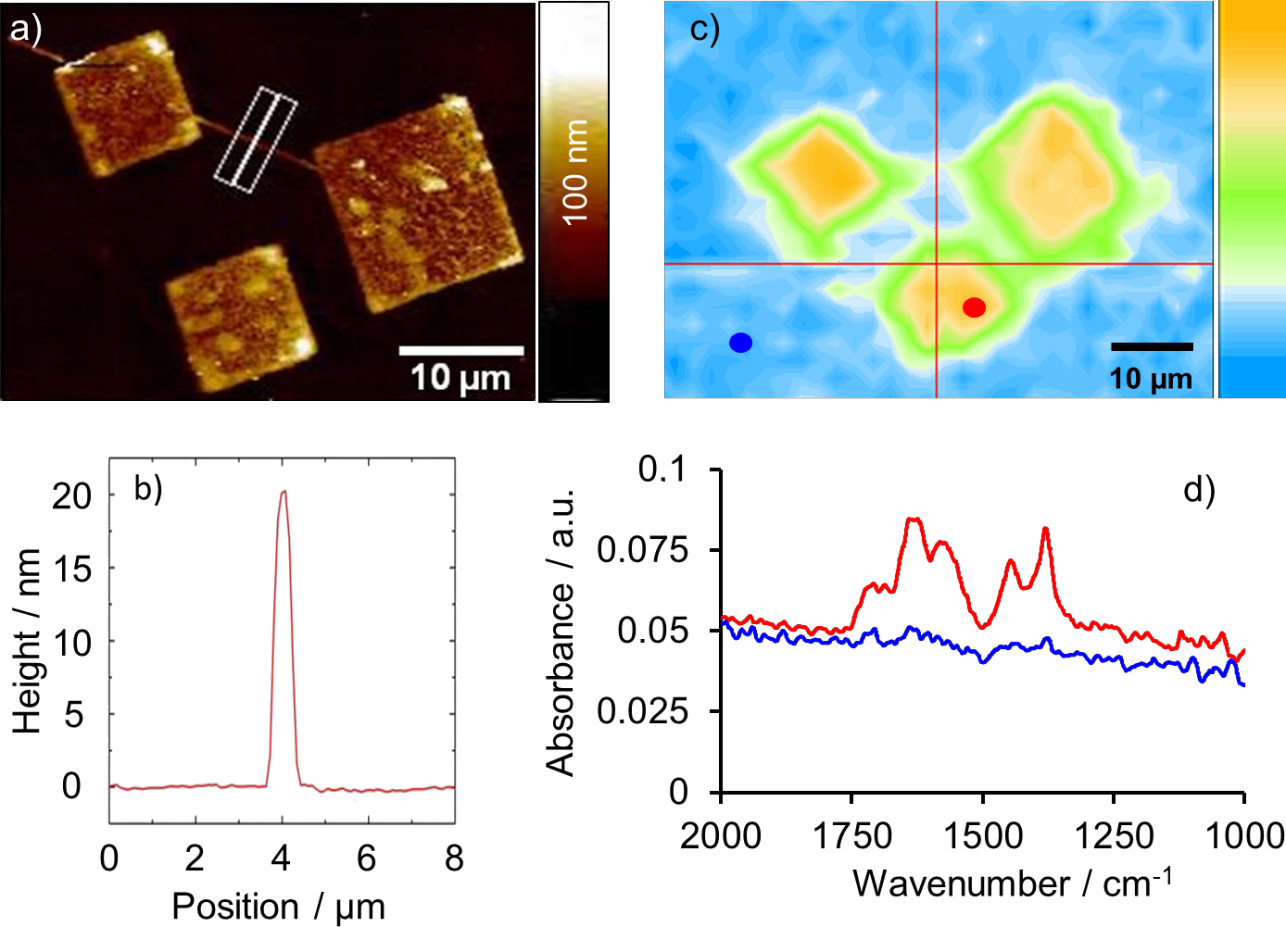
(a) AFM-topography image of HKUST-1 structures, prepared by spray coating after grafting squared areas of MPA into the DT matrix thiol. (b) Cross section along the white line of (a). (c) FT-IR imaging of the wavenumber region between 1510 cm^−1^ and 1780 cm^−1^. (d) FT-IR spectra representing the area inside the squares (red line in (d) corresponding to red dot in (c)) and outside of the structures (blue line in (d), according to the blue dot in (c)).

After characterizing the nanografted features in some detail, the nanografted patterns were used as a substrate for the SURMOF deposition. Figure 5a shows the AFM topography images of a surface area, which was first patterned by grafting three squares of MPA into a DT SAM layer, followed by the layer-by-layer growth of HKUST-1 SURMOF using the spray method. The SURMOF structures in Figure 5a correspond to the elevated structures. The micrographs demonstrate that the SURMOF growth is strictly limited to the grafted areas and no unspecific deposition of the HKUST-1 can be recognized outside of the squares. When the lithography program for the grafting experiment starts, the AFM tip moves to its initial position, while the tip is still in contact with the surface. This results in the grafting of the MPA SAM into the DT matrix SAM along this trace. Therefore, a line consisting of HKUST-1, which connects the big square with one of the small squares can be recognized beside the laminar SURMOF structures in Figure 5a. This line was used to determine the height of the grown SURMOF layer, because the laminar structures show an inhomogeneous height distribution. The evaluation of the cross section of this SURMOF line (Figure 5b) along the white line in Figure 5a yields a height of 20 nm which is, according to the height scale, comparable to the height of those SURMOF areas grown with a lower layer thickness. From Figure 5a it can be clearly seen that there are some areas, which are significantly higher than 20 nm.

To demonstrate that the deposited material consists of HKUST-1, FT-IR imaging of the surface area was carried out using an ATR FT-IR microscope. Figure 5c shows the lateral distribution of the band intensities between 1510 cm^−1^ and 1780 cm^−1^, which includes the typical region for the asymmetric stretching vibrations of carboxylate groups. It is obvious that the highest intensity can be recognized on the grafted areas as expected. Figure 5d shows two spectra, which represent the grafted areas (red spot in Figure 5c and red line in Figure 5d) and the surrounding area (blue spot in Figure 5c and blue line in Figure 5d) after the growth of HKUST-1, respectively. The typical bands of the asymmetric –COO stretching vibrations of the carboxylate groups can be detected between 1610 cm^−1^ and 1550 cm^−1^, while the bands between 1420 cm^−1^ and 1300 cm^−1^ represent the symmetric –COO vibration bands of the deprotonated linker [33–34]. The band at 1446 cm^−1^ corresponds to the C–C vibration of the aromatic ring of the linker molecule [35]. The bands between 1730 cm^−1^ and 1680 cm^−1^ can be assigned to the stretching vibration of the –CO group and the C–OH group of the protonated SAM and some residual BTC linker molecules in the SURMOF structures. These findings demonstrate the selective growth of HKUST-1 SURMOF on the grafted areas.

### HKUST-1 SURMOF on MHDA nanografted structures

**Figure 6:**
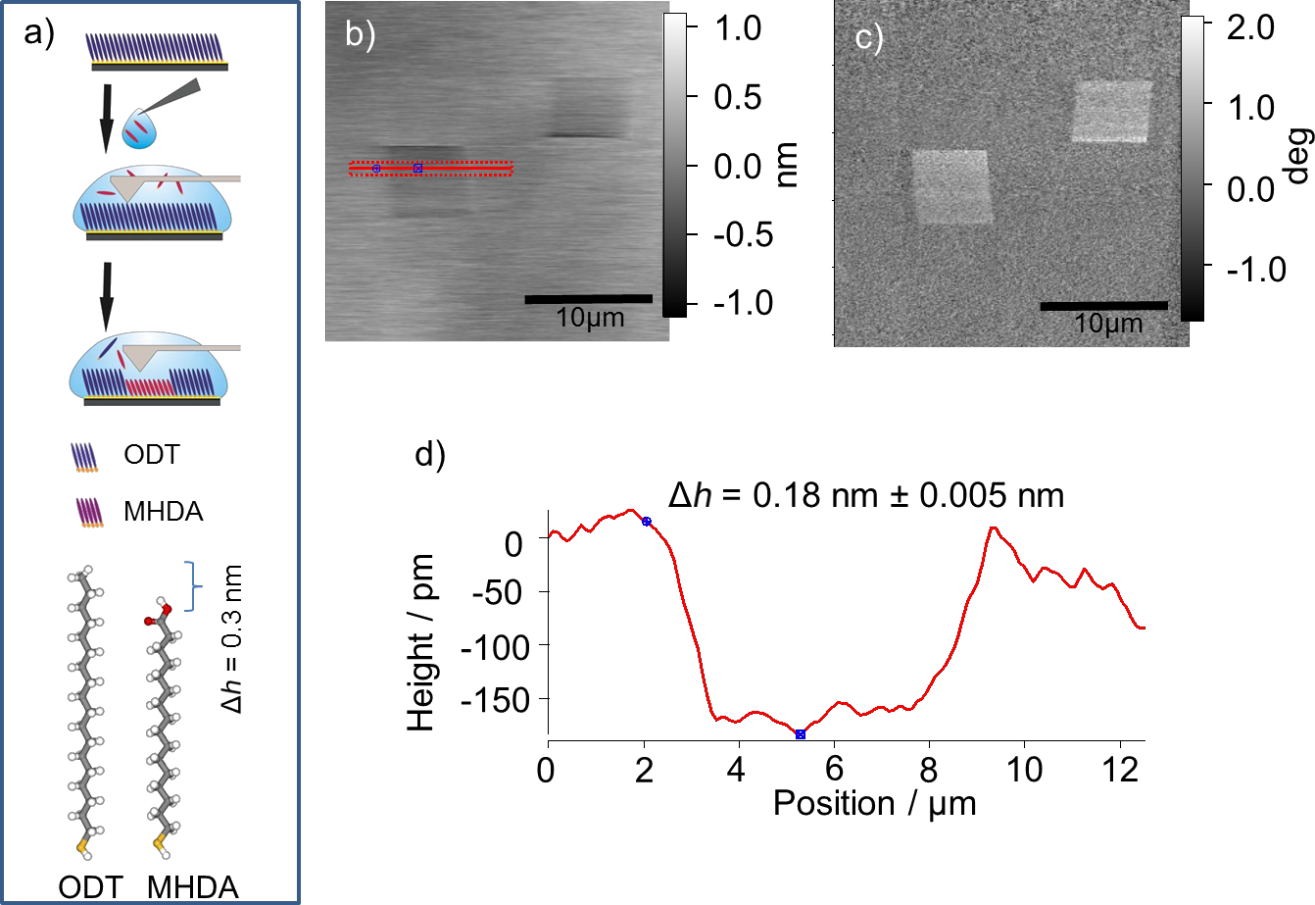
(a) Scheme of the grafting experiment and used SAM molecules. AFM-topography (b) and -phase (c) images of the MHDA areas grafted into an ODT matrix SAM. (d) Cross section along the red line in (b).

As a second thiol supporting the selective growth of SURMOF structures, 16-mercaptohexadecanoic acid (MHDA), was used and grafted into a matrix SAM made from 1-octadecanethiol (ODT, Figure 6a). In this case ODT was used because the back bone length of this thiol is comparable to that of the grafted MHDA. A matrix SAM consisting of the significantly shorter 1-decanethiol (DT), as it was used for the grafting of 3-mercaptopropionic acid (MPA), would be spontaneously substituted by the longer MHDA molecules over time.

In Figure 6b and Figure 6c the AFM topography and AFM phase contrast images of the MHDA structures grafted into the ODT SAM are displayed, respectively. The grafted regions appear as dark areas in the topography image, indicating a depression. The significant phase contrast is a hint that the grafted areas are coated with molecules, which have a chemical termination different from that of the surrounding SAM regions. According to the cross section of Figure 6d measured along the red line in Figure 6b, the average height difference between the matrix SAM and the grafted MHDA area amounts to about 0.2 nm. This value is in good agreement with the theoretical length difference of approx. 0.2 nm between the MHDA and the ODT molecule.

**Figure 7:**
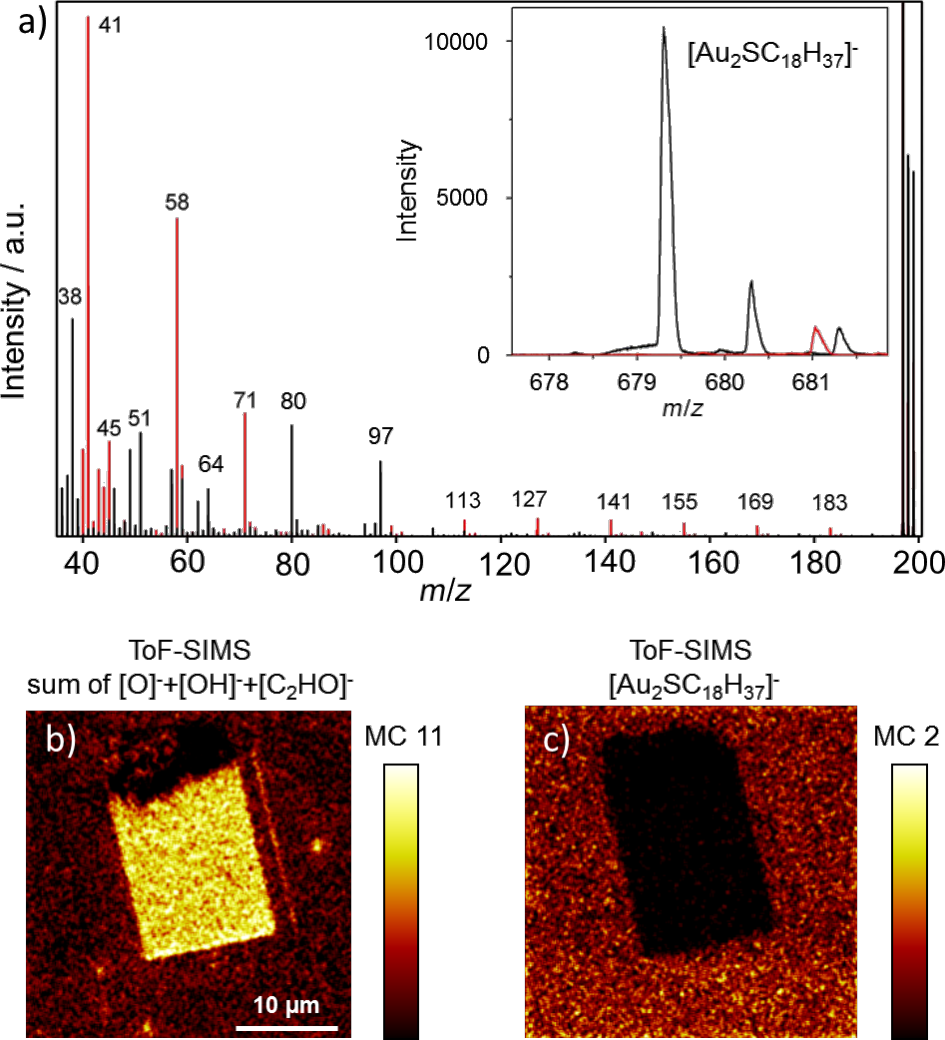
(a) ToF-SIMS spectra of ODT (black) and MHDA (red) SAMs on gold substrates. The inset shows the [Au_2_SC_18_H_37_]^−^ peak at *m*/*z* 679 characteristic for ODT. In (b) and (c) the mapping of the [O]^−^ + [OH]^−^ + [C_2_HO]^−^ and the [Au_2_SC_18_H_37_]^−^ (*m*/*z* 679) fragments, respectively, are shown, demonstrating the successful grafting of MHDA into the ODT SAM.

To characterize the chemical composition of the structured areas, ToF-SIMS measurements were carried out. Characteristic ions of ODT and MHDA are detected at *m*/*z* 483, [AuSC_18_H_38_]^−^, *m*/*z* 679, [Au_2_SC_18_H_37_]^−^, and *m*/*z* 681, [Au_2_SC_16_H_31_O_2_]^−^. Apart from these characteristic pseudo-molecular peaks the comparison of small fragments providing strong SIMS signals allows for differentiation between the two thiols. The results of this investigation are summarized in Figure 7a, in which the ToF-SIMS spectra of the matrix thiol (ODT) and the grafted thiol (MHDA) are superimposed. In case of MHDA (red line) the following peaks are identified: *m*/*z* 41, [C_2_HO]^−^, *m*/*z* 58, [C_2_H_2_O_2_]^−^, and *m*/*z* 71, [C_3_H_3_O_2_]^−^. In addition there are several [(CH_2_)*_n_*C_3_H_3_O_2_]^−^ fragments with *n* = 1–8 (*m*/*z* 85, 99, 113, 127, 141, 155, 169, 183), as well as [O]^−^ and [OH]^−^. In case of the ODT SAM, peaks marked with *m*/*z* 64, 80, and 97 in the ODT spectrum can be assigned to [SO_2_]^−^, [SO_3_]^−^, [SO_4_H]^−^, respectively. These ions can be found for some thiol SAMs with oxidized thiol groups [36]. The extraordinary high ionization yield of these ions results in strong SIMS signals but provides no quantitative information. A mapping of the lateral distribution of the characteristic masses of MHDA and ODT results in Figure 7b and Figure 7c, respectively. The high intensity of the *m*/*z* 41 fragments (which are assigned to [C_2_HO]^−^) in Figure 7b demonstrates that the grafting was successfully carried out.

**Figure 8:**
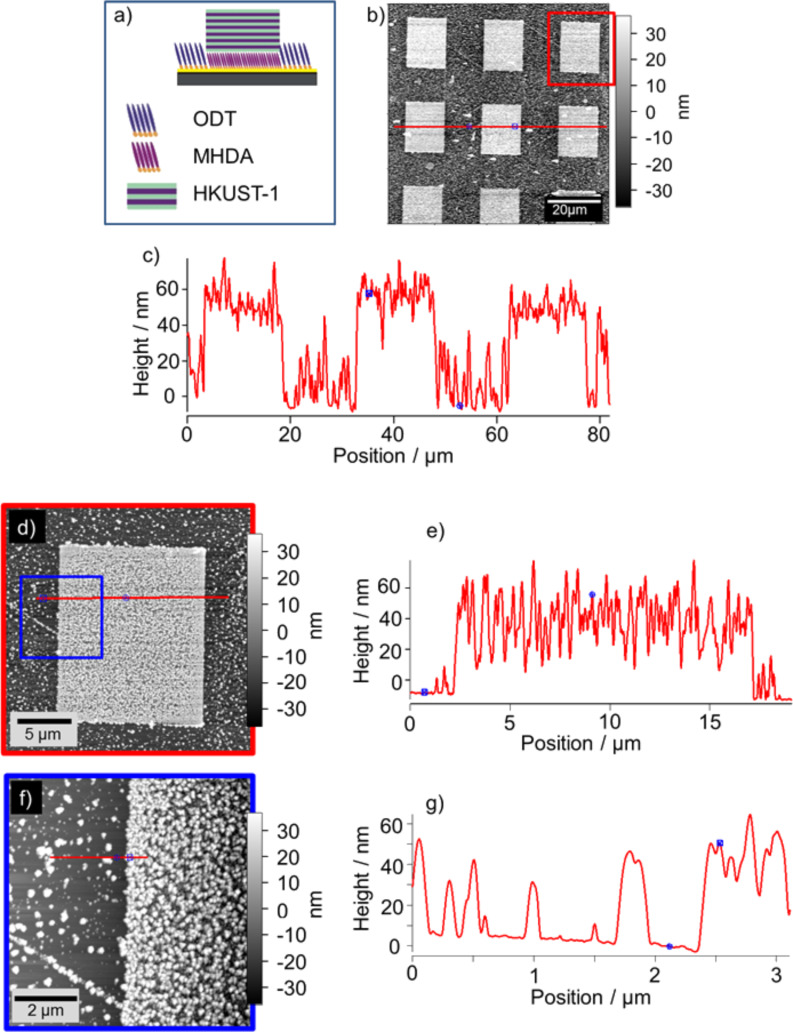
(a) Scheme of the layer cross-section of the AFM topography image of rectangular HKUST-1 structures site-selectively grown on the grafted MHDA areas. (b) AFM topography image of an array of rectangular HKUST-1 structures, grown on the MHDA terminated areas. (c) Cross section along the red line in (b). (d) and (f) detailed AFM topography images of the structure shown in (b) with the corresponding cross sections in (e) and (g), respectively.

After demonstrating the successful grafting of MHDA, the deposition of the HKUST-1 SURMOF was carried out again by employing the spray method. In Figure 8a the structure of the layers are displayed schematically. The corresponding AFM topography image of the rectangular SURMOF structures is presented in Figure 8b. According to the cross section in Figure 8c along the red line of Figure 8b, the average height of the SURMOF structures amount to about 63 nm. Interestingly, this value is significantly larger than that measured for the SURMOF-structures grown on the MPA grafted areas (Figure 5). Note that in both cases the same number of deposition cycles (40) has been applied.

From the more detailed AFM-topography images of Figure 8d and Figure 8f, a particulate-like growth of the SURMOF material can be observed on top of both the MHDA-terminated, grafted areas and the matrix SAM. A higher density of crystallites can be found at the MHDA terminated surface areas. This is in contrast to the findings for the selective SURMOF grown on the MPA-terminated areas grafted in the DT SAM where no particulate material can be detected on the OD SAM (Figure 5). A roughness (RMS) of 28.9 ± 3.9 nm was determined for the MHDA-based structures, which is slightly rougher than the surrounding ODT SAM based areas with a roughness of 24.3 ± 3.1 nm. The cross sections in Figures 8e (along the red line in Figure 8d) and 8g (along the red line in Figure 8f) exhibit a comparable height of the crystals irrespective of the SAM on which they grew. The fact that the growth of crystals can be observed on the ODT SAM is a hint that a spontaneous exchange between the ODT and the MHDA took place locally, and that this thiol combination is not as advantageous as the grafting of MPA into a matrix SAM of DT.

## Conclusion

In the present proof-of-concept study it has been demonstrated that the selective growth of micro- and sub-microsized MOF-structures can be achieved by combining layer-by-layer growth with AFM-based nanografting. As the amount of available SURMOF structures is limited by the applied nanografting method and the structure dimensions are in the micrometer-range, the oriented growth of this structures has not yet been proven (e.g., by XRD). This will be a future challenge. The results also show, that an appropriate combination of thiols that activate the SURMOF growth and thiols that set up the matrix must be figured out, in order to avoid an undesirable and uncontrolled growth of SURMOF crystallites on the non-grafted areas. (This was observed for the SURMOFs based on MHDA grafted into an ODT matrix SAM.) Nevertheless the availability of such MOF-structures opens a wide range of opportunities to create three-dimensional structures of highly porous materials, which offer a high flexibility with regard to the geometrical shape, size and height. It has been demonstrated in previous works that the direction of the oriented growth of the SURMOFs can be influenced by the chemical termination of the supporting SAM [14–15] and that the orientation of the SURMOF structures in turn influences the adsorption of guest molecules in the MOF host-structure [37]. To create a pattern with differently oriented SURMOF structures on the same substrate, which will be interesting for biological application, would require a technique, which allows the patterning of the substrate with different SAMs. Although usual structuring methods, such as micro contact printing or e-beam lithography, can be used for SAM patterning as well, those techniques reach a limit when patterns with differently terminated SAMs on the same substrate have to be prepared. This is because either the samples have to be removed from the UHV and/or a realignment of the sample as well as the recovery of the formerly prepared pattern becomes necessary, before the next structuring step can be carried out. By contrast, in the case of AFM nanografting the sample stays in the liquid cell and differently terminated SAM structures can be written just by exchanging the supernatant thiol solution. Although the grafting technique will not be suitable for a high throughput and large scale preparation of substrates, it has the power as scientific tool to prepare highly specific substrates for basic research.

## Experimental

### Sample preparation

For substrate preparation and surface functionalization we followed a previously described route [38]. Briefly, Au coated substrates for the nanografting experiments were prepared by depositing a 5 nm layer of titanium and subsequently 130 nm of gold onto polished [100] silicon wafers (Siegert Wafer, Germany) using a Leybold Univac evaporator (Leybold Optics, Germany). Metal deposition was done at room temperature at a base pressure of 10^−7^ bar. For the preparation of the initial SAMs on the gold covered silicon wafer 1-octadecanethiol (ODT) or 1-decanethiol (DT) were used. The SAM was produced by immersing the substrate in a 1 mM ethanolic solution of ODT or DT for 12 h.

### Nanografting and AFM characterization

All AFM investigations were carried out with a MFP-3D Bio AFM (Asylum Research, Mannheim). Grafting experiments and AFM investigation of the surface were performed in a polystyrene petri dish (BD falcon, VWR, Germany) mounted on the scanner of the MFP-3D Bio. For grafting experiments the “B” tips of NSC-35 cantilever chips (Micromash, Germany) with the nominal force constant of 16 N/m were used. Both the nanografting of 3-mercaptopropionic acid (MPA) in a DT SAM and the nanografting of 16-mercaptohexadecanoic acid (MHDA) in ODT SAM were performed in a 0.2 mM ethanolic solution of the corresponding acid. In both cases 10% (v/v) glacial acetic acid was added to the grafting solutions. The nanografting in the case of MPA/DT was performed in contact mode with a loading force of 345 nN, while for MHDA/ODT a loading force of 303 nN was used to remove thiols out of the existing matrix SAM. All AFM surface analysis experiments were carried out in the intermittent contact mode and the AFM tip was scanned at an angle of 90° relative to the longitudinal axis of the cantilever in several scan ranges. The AFM was used in a closed loop on all three axes. AFM images were evaluated with the IGOR software.

### SURMOF preparation

**Figure 9:**
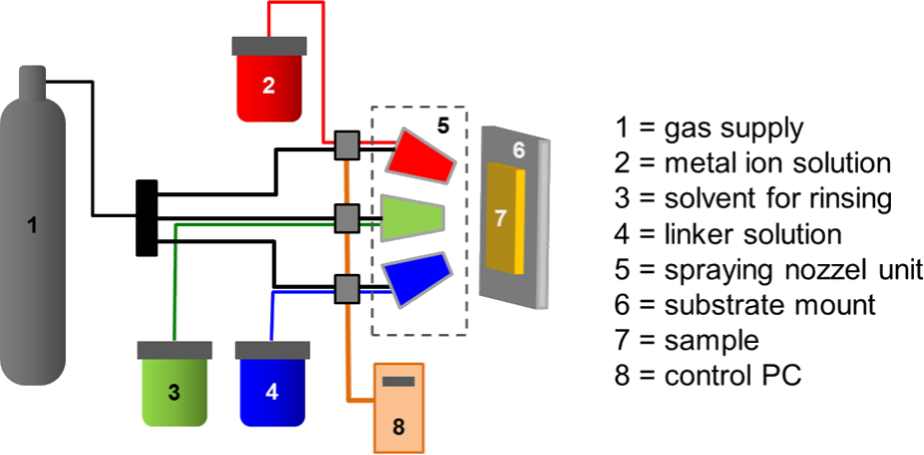
Setup employed for the fabrication of MOF thin films with the spray method [39].

For the preparation of the SURMOF on the grafted areas a new, recently published LBL spray method was used [39]. In Figure 9 the scheme of the spray method for SURMOF preparation is shown. The horizontally mounted sample is subsequently sprayed (1) with the ethanolic Cu acetate solution (*c* = 1.0 mM / 10 s spraying time / 20 s waiting time), (2) with pure ethanol for rinsing (3 s spraying time / 1 s waiting time), (3) with the linker solution consisting of an ethanolic solution of 1,3,5-benzenetricarboxylic acid (*c* = 0.1 mM / 15 s spraying time / 20 s waiting time) and (4) finally again with pure ethanol (3 s spraying time / 1 s waiting time). This procedure is repeated 40 times. At the end of this process the sample was rinsed again with pure ethanol for 2 s.

### FT-IR-imaging

FT-IR-imaging was performed on a Bruker Hyperion 3000 FT-IR (Bruker Optics, Ettlingen, Germany) imaging system equipped with a 20× ATR objective with a Ge-Crystal tip. A 64 × 64 pixel FPA detector sensitive to a range of 900–3800 cm^−1^ was used. 4096 spectra were aquired in one measurement over a field of view of 32 × 32 µm, 4 × 64 scans were collected over an area of 64 × 64 µm with a spectral resolution of 4 cm^−1^.

### ToF-SIMS

Time-of-ﬂight secondary ion mass spectrometry was performed on a TOF-SIMS 5 instrument from ION-TOF GmbH, Münster, Germany. This instrument is equipped with a Bi cluster liquid metal ion source and a reﬂectron type time-of-ﬂight analyzer. For spectrometry short primary ion pulses (<1 ns) of Bi_1_^+^ and Bi_3_^+^ at an energy of 25 keV were applied providing high mass resolution secondary ion spectra with a moderate spot size of about 5 mm (bunched mode). Spectrometry was performed in static SIMS mode by limiting the primary ion dose to <10^11^ ions/cm^2^. High lateral resolution images were acquired in a primary ion source mode providing a lateral resolution of about 200 nm with nominal mass resolution (burst alignment mode). No charge compensation was required. Spectra were calibrated on omnipresent C^−^, CH^−^, CH_2_^−^, Au^−^, and molecular peaks.
